# Examining the role of systemic chronic inflammation in diet and sleep relationship

**DOI:** 10.1177/02698811221112932

**Published:** 2022-07-21

**Authors:** Piril Hepsomali, John A Groeger

**Affiliations:** 1 School of Psychology, University of Roehampton, London, UK; 2 Unilever R&D, Colworth Science Park, Bedford, UK; 3 Department of Psychology, School of Social Sciences, Nottingham Trent University, Nottingham, UK

**Keywords:** Diet, sleep, blood cells, C-reactive protein, inflammation

## Abstract

**Background:**

It is well known that systemic chronic inflammation (SCI), which can be modulated by diet, is associated with poor sleep outcomes. However, the role of SCI in diet health and sleep quality relationship has not been well established.

**Methods:**

Here, by using the UK Biobank data set, we assessed the association between markers of SCI (leukocyte, platelet, lymphocyte, neutrophil, and basophil counts; C-reactive protein levels and neutrophil to lymphocyte ratio (NLR)), habitual intake of food groups, diet health and sleep quality in 449,084 participants. We also formally tested the possibility that SCI might mediate the relationship between diet health and sleep quality.

**Results:**

Our results revealed (i) negative associations between SCI and food groups that are abundant in healthy diets (fruit, vegetable and oily and non-oily fish) and (ii) positive associations between SCI and food groups that are abundant in unhealthy diets (processed meat). Sleep quality was also negatively associated with platelet counts, CRP levels and NLR. Crucially, however, while platelet and neutrophil counts, CRP levels and NLR fully mediated the association between diet health and sleep quality, leukocyte, lymphocyte and basophil counts partially mediated the associations between diet health and sleep quality.

**Conclusion:**

Reducing SCI via dietary interventions could be an effective primary and/or complementary strategy to increase sleep quality. Further interventional trials are warranted to (i) establish the strength of associations, preferably by using validated diet and sleep measures and (ii) examine longer term effects of anti-inflammatory diets on sleep-, diet- and inflammation-related health outcomes.

## Introduction

It is well known that some nutrients, nutritional deficiencies or abundances and food items may affect sleep (Doherty et al., 2019). Both small experimental and large observational studies have shown benefits of large quantities of carbohydrates and fat, some proteins (amino acid tryptophan), group B vitamins, magnesium as well as foods containing tryptophan, melatonin and phytonutrients (e.g. cherries, kiwifruit, milk) on various sleep outcomes (see reviews, Peuhkuri et al., 2012; Sanlier and Sabuncular, 2020; St-Onge et al., 2016). On the other hand, following a shift in nutritional psychiatry’s research scope towards food group and dietary pattern analysis (Hu, 2002), recent studies have shown that the Mediterranean (MED)-style diets (Godos et al., 2019) and diet health/quality (Hepsomali and Groeger, 2021) were associated with better sleep quality.

MED-style/healthy diets and food groups that are abundant in these diets (such as fruits, vegetables, wholegrains and seafood) are known to contain significant amounts of fibre, polyphenols and omega-3 poly-unsaturated fatty acids. These nutrients have anti-inflammatory, neuroprotective and prebiotic properties (González et al., 2011; Widmer et al., 2015) and also are associated with reduced systemic inflammation markers, such as the C-reactive protein (CRP) (Ma et al., 2008; Sureda et al., 2018; Wisnuwardani et al., 2020). Additionally, systemic chronic inflammation (SCI) biomarkers were also found to be associated with diet. For instance, it has been shown that leukocyte count and neutrophil to lymphocyte ratio (NLR) were inversely associated with adherence to the MED-style diets (Bonaccio et al., 2014; Rodríguez-Rodríguez et al., 2020) and higher intake of vegetables (Menni et al., 2021). Similarly, lower platelet count has also been associated with adherence to the MED-style diets (Bonaccio et al., 2014).

There is also growing evidence that SCI is associated with sleep. Observational studies showed associations between (i) higher levels of CRP and interleukin-6 and shorter sleep duration (⩽5 h/night) (Ferrie et al., 2013) and (ii) higher leukocyte counts and shorter sleep duration (<8 h/night) (Pérez de Heredia et al., 2014). Similarly, intervention studies found that long-term sleep restriction (5 days of restricted/shortened sleep, 4 h/night: 03.00–07.00 h) increased total white blood cell, monocytes, neutrophils and lymphocytes count (Lasselin et al., 2015) and both acute total and short-term partial sleep deprivation increased CRP concentrations (Meier-Ewert Hans et al., 2004). In terms of sleep quality, although there is some observational evidence showing associations between higher CRP levels and lower sleep quality (Lee et al., 2020; Liu et al., 2014), research is limited for other SCI markers albeit suggestive of a similar relationship (e.g. Nishitani and Sakakibara, 2007; Obayashi et al., 2016).

Sleep-SCI relationship is bidirectional (Besedovsky et al., 2019) and both poor sleep and high levels of SCI are linked to various health outcomes including diabetes (Grandner et al., 2016; Tsalamandris et al., 2019), obesity (Ellulu et al., 2017; Marshall et al., 2008) and even mortality (Cappuccio et al., 2010; Proctor et al., 2015). These same health outcomes are, of course, affected by diet quality (Ley et al., 2016; Neelakantan et al., 2018; Wolongevicz et al., 2010).

Understanding the interplay between diet, SCI and sleep would enable us to establish priorities for anti-inflammatory dietary guidelines that may help self-management and/or treatment of sleep challenges and to prevent the development and/or progression of diet, SCI and sleep-related long-term health outcomes. Therefore, in the current study, by utilising the large UK Biobank (UKB) data set, we examined the roles of (i) dietary intake in predicting SCI biomarkers, (ii) SCI biomarkers in predicting sleep quality and (iii) SCI biomarkers in diet–sleep relationship.

## Method

This study (a part of the UKB project 61818) utilised the UKB data set (Sudlow et al., 2015). For the UKB study, ethical approval was granted by the Northwest Multi-Centre Ethics committee (ref: 11/NW/0382). This study was performed in accordance with these guidelines and regulations, under the UKB ethics governance and framework (https://www.ukbiobank.ac.uk/ethics/).

### Study population

Detailed study design and methods of the UKB study have been reported elsewhere (Sudlow et al., 2015). Briefly, the UKB study recruited more than 500,000 adults with the age of 40–69 years between 2006 and 2010. At their initial visit, participants provided a written informed consent and completed a touch screen questionnaire that assessed various sociodemographic, lifestyle and health behaviour variables, including food intake, sleep and also provided biological samples such as blood biomarkers (see the Supplemental Methods for detailed information about the recruitment procedure).

### Measures

In the current study, we used responses from the UKB’s food frequency, sleep and psychological factors and mental health questionnaires at baseline to create various scores including healthy diet, mental health symptomatology and problematic sleep index (see the Supplemental Methods for detailed descriptions). Data fields of interest and the methods for calculating healthy diet and mental health symptomatology scores are available elsewhere (Hepsomali and Groeger, 2021). The Problematic sleep index (Groeger and Hepsomali, under review) is a compositive measure of self-reported sleep problems, such as sleep duration abnormal for a participant’s age, delayed sleep onset, difficulty waking, overnight wakefulness, snoring and so on, where higher scores indicate fewer problems and hence better sleep. Similar to our previous study (Hepsomali and Groeger, 2021), we also utilised total food group intake scores (vegetable, fruit, oily fish, non-oily fish, unprocessed red meat and processed meat). Finally, various blood assay results including leukocyte (10^9^ cells/L; Data Field: 30000), platelet (10^9^ cells/L; Data Field: 30080), lymphocyte (10^9^ cells/L; Data Field: 30120), neutrophil (10^9^ cells/L; Data Field: 30140) and basophil (10^9^ cells/L; Data Field: 30160) counts as well as levels of CRP (mg/L; Data Field: 30710) were included in the study. We also calculated NLR.

### Statistical analyses

All analyses were performed in IBM SPSS Statistics 26.0.0.0. Questionnaire response options, ‘do not know’ or ‘prefer not to answer’, were handled as missing values.

First, bivariate Pearson correlations (two-tailed, with adjusted *p*-value for multiple testing using the Benjamini–Hochberg method) between all measures were examined to evaluate the presence of anticipated relationships between diet, sleep and SCI biomarkers.

Second, to quantify the associations between healthy diet score and SCI biomarkers, separate linear regression analyses were performed. In the unadjusted models, the associations between food group intake (vegetable, fruit, oily fish, non-oily fish, unprocessed red meat and processed meat) and biomarkers of SCI were analysed. In the adjusted models, age, sex, body mass index (BMI), total number of mental health symptoms reported, Townsend Deprivation Index scores (as a marker of socioeconomic status (SES)) and overall health ratings were added as covariates in the linear models described above.

Third, we also performed separate unadjusted and adjusted (age, sex, BMI, mental health symptomatology, SES and overall health ratings) linear regression analyses to examine the role of biomarkers of SCI on problematic sleep index.

Finally, in separate covariate adjusted (age, sex, BMI, mental health symptomatology, SES and overall health ratings) mediation models, we examined whether SCI biomarkers mediate the relationship between healthy diet score and the problematic sleep index with Process macro (http://processmacro.org/index.html) in SPSS (Hayes, 2017) using 5000 bootstrap resamples and 95% confidence intervals. Standardised coefficients are reported throughout.

## Results

Baseline characteristics of 449,084 participants according to leukocyte, platelet, lymphocyte, neutrophil and basophil counts; CRP levels and NLR are presented in the Supplemental Results Table 1.

### Associations between diet, sleep and SCI biomarkers

As seen in Table 1, problematic sleep index scores showed negative significant associations with all SCI biomarkers (i.e. less SCI is associated with less problematic sleep). Similarly, healthy diet score was negatively associated with all SCI biomarkers except the platelet count. Moreover, problematic sleep index and healthy diet score was positively correlated. Using a split-half approach, consistent with the results from the total data set, all but one correlation (healthy diet and platelet count) was found to be statistically significant in each half; thus, both halves of the data set produce similar results (see Supplemental Results Table 2).

**Table 1.** Correlation matrix showing the relationships between problematic sleep index, healthy diet score and SCI biomarkers.

		1	2	3	4	5	6	7	8	9
1. Problematic sleep index	*r*	1	0.016***	−0.053***	−0.039***	−0.021***	−0.056***	−0.018***	−0.081***	−0.021***
	*N*		410,217	393,878	393,881	393,153	393,153	393,153	386,054	393,148
2. Healthy diet score	*r*		1	−0.072***	0.002	−0.007***	−0.086**	−0.011***	−0.051***	−0.049***
	*N*			478,149	478,151	477,265	477,265	477,265	468,550	477,258
3. Leukocyte count	*r*			1	0.205***	0.706***	0.794***	0.250***	0.188***	0.204***
	*N*				478,145	477,265	477,265	477,265	456,520	477,258
4. Platelet count	*r*				1	0.072***	0.226***	0.075***	0.124***	0.036***
	*N*					477,261	477,261	477,261	456,522	477254
5. Lymphocyte count	*r*					1	0.154***	0.184***	0.021***	−0.275***
	*N*						477,265	477,265	455,690	477,258
6. Neutrophil count	*r*						1	0.149***	0.240***	0.535***
	*N*							477,265	455,690	477,258
7. Basophil count	*r*							1	0.052***	−0.018***
	*N*								455,690	477,258
8. C-reactive protein	*r*								1	0.160***
	*N*									455,683
9. NLR	*r*									1
	*N*									

*** Correlation is significant with a false discovery rate <0.001 level (two-tailed).

### Food group intake predicts SCI biomarkers

Results from the regression models are presented in Table 2.

**Table 2.** Regression analysis summary for SCI biomarkers.

	Unadjusted													
	Leukocyte count		Platelet count		Lymphocyte count		Neutrophil count		Basophil count		C-reactive protein		NLR	
	*β* (SE)	LL UL	*β* (SE)	LL UL	*β* (SE)	LL UL	*β* (SE)	LL UL	*β* (SE)	LL UL	*β* (SE)	LL UL	*β* (SE)	LL UL
Constant	*** (0.012)	6.823 6.870	*** (0.340)	265.818 267.151	*** (0.007)	1.917 1.943	*** (0.008)	4.235 4.266	*** (0.000)	0.036 0.037	*** (0.025)	2.471 2.568	*** (0.007)	2.385 2.414
Vegetable	−0.001 (0.001)	0.003 0.001	−0.008*** (0.028)	−0.204 −0.096	0.006*** (0.001)	0.001 0.003	−0.005** (0.001)	−0.003 −0.001	0.002 (0.000)	0.000 0.000	−0.006*** (0.002)	−0.012 −0.004	−0.009*** (0.001)	−0.005 −0.002
Fruit	−0.042*** (0.001)	−0.037 −0.032	−0.013*** (0.036)	−0.377 −0.236	−0.005*** (0.001)	−0.004 −0.001	−0.053*** (0.001)	−0.030 −0.027	−0.014*** (0.000)	0.000 0.000	−0.027*** (0.003)	−0.050 −0.040	−0.026*** (0.001)	−0.014 −0.011
Unprocessed red meat	0.018*** (0.002)	0.019 0.026	−0.008*** (0.054)	−0.361 −0.149	0.012*** (0.001)	0.006 0.010	0.014*** (0.001)	0.009 0.014	−0.011*** (0.000)	0.000 0.000	0.030*** (0.004)	0.066 0.081	−0.003* (0.001)	−0.005 0.000
Processed meat	0.048*** (0.003)	0.091 0.103	−0.048*** (0.091)	−2.898 −2.541	−0.002 (0.002)	−0.006 0.001	0.059*** (0.002)	0.075 0.084	0.007*** (0.000)	0.000 0.000	0.020*** (0.007)	0.068 0.094	0.044*** (0.002)	0.049 0.057
Oily fish	−0.034*** (0.004)	−0.085 −0.070	−0.053*** (0.106)	−3.650 −3.235	−0.003* (0.002)	−0.008 0.000	−0.046*** (0.002)	−0.075 −0.065	−0.018*** (0.000)	−0.001 −0.001	−0.022*** (0.008)	−0.117 −0.087	−0.023*** (0.002)	−0.036 −0.027
Non-oily fish	0.000 (0.004)	−0.007 0.010	−0.001 (0.123)	−0.349 0.134	0.005** (0.002)	0.003 0.012	−0.002 (0.003)	−0.010 0.001	−0.001 (0.000)	0.000 0.000	−0.002 (0.009)	−0.028 0.007	−0.008*** (0.003)	−0.018 −0.008
	Adjusted^#^													
Constant	*** (0.048)	4.455 4.570	*** (0.822)	275.579 278.800	*** (0.017)	1.315 1.380	*** (0.020)	2.778 2.855	*** (0.001)	0.027 0.030	*** (0.059)	−5.285 −5.052	*** (0.018)	2.018 2.088
Vegetable	−0.004*** (0.001)	−.004 .000	−0.012*** (0.027)	−0.267−0.162	0.003* (0.001)	0.000 0.002	−0.006*** (0.001)	−0.004 −0.001	0.001 (0.000)	0.000 0.000	−0.011*** (0.002)	−0.019 −0.011	−0.006*** (0.001)	−0.003 −0.001
Fruit	−0.037*** (0.001)	−.033 −.028	−0.021*** (0.035)	−0.560 −0.423	−0.006*** (0.001)	−0.004 −0.001	−0.047*** (0.001)	−0.027−0.024	−0.014*** (0.000)	0.000 0.000	−0.025*** (0.003)	−0.046 −0.036	−0.021*** (0.001)	−0.012 −0.009
Unprocessed red meat	0.010*** (0.002)	.008 .016	0.001 (0.053)	−0.071 0.136	0.008*** (0.001)	0.003 0.007	0.008*** (0.001)	0.004 0.009	−0.009** (0.000)	0.000 0.000	0.012*** (0.004)	0.023 0.038	−0.004** (0.001)	−0.005 −0.001
Processed meat	0.034*** (0.003)	.061 .074	0.007*** (0.092)	0.241 0.600	0.004** (0.002)	0.001 0.008	0.043*** (0.002)	0.054 0.062	0.012*** (0.000)	0.000 0.001	0.008*** (0.007)	0.021 0.047	0.023*** (0.002)	0.023 0.031
Oily fish	−0.029*** (0.004)	−.074 −.060	−0.040*** (0.104)	−2.808 −2.401	−0.000 (0.002)	−0.004 0.004	−0.041*** (0.002)	−0.067 −0.057	−0.014*** (0.000)	−0.001 −0.001	−0.018*** (0.008)	−0.097 −0.067	−0.027*** (0.002)	−0.041 −0.032
Non-oily fish	0.000 (0.004)	−.010 .007	−0.008*** (0.120)	−0.821 −0.352	0.003 (0.002)	−0.001 0.009	−0.002 (0.003)	−0.010 0.001	−0.001 (0.000)	0.000 0.000	−0.006*** (0.009)	−0.052 −0.018	−0.005** (0.003)	−0.014 −0.003
Age	0.033*** (0.000)	.007 .009	−0.053*** (0.011)	−0.413 −0.370	0.010*** (0.000)	0.001 0.002	0.027*** (0.000)	0.004 0.005	−0.005** (0.000)	0.000 0.000	0.055*** (0.001)	0.028 0.031	0.036*** (0.000)	0.005 0.006
Sex (F = 0/M = 1)	−0.013*** (0.007)	−.090 −.064	−0.241*** (0.182)	−29.377 −28.663	−0.057*** (0.004)	−0.141 −0.127	−0.010** (0.004)	−0.036 −0.019	−0.044*** (0.000)	−0.005 −0.004	−0.061*** (0.013)	−0.553 −0.501	0.074*** (0.004)	0.180 0.196
BMI	0.147*** (0.001)	.051 .053	0.038*** (0.019)	0.444 0.518	0.074*** (0.000)	0.018 0.019	0.094*** (0.000)	0.027 0.029	0.022*** (0.000)	0.000 0.000	0.219*** (0.001)	0.196 0.202	−0.044*** (0.000)	−0.013 −.011
MH symp. (higher: worse)	0.020*** (0.001)	−.007 −.003	0.003 (0.028)	−0.008 0.102	−0.007*** (0.001)	−0.004 −0.001	−0.004* (0.001)	−0.003 0.000	−0.004* (0.000)	0.000 0.000	−0.010*** (0.002)	−0.018 −0.010	−0.005** (0.001)	−0.003 −0.001
Health rating (Higher: worse)	0.101*** (0.005)	.284 .302	0.27*** (0.129)	1.963 2.468	0.25*** (0.003)	.036 0.046	0.116*** (0.003)	0.219 0.232	0.038*** (0.000)	0.002 0.003	0.095*** (0.009)	0.550 0.587	0.084*** (0.003)	0.141 0.152
SES (higher: least affluent)	0.039*** (0.001)	.025 .029	0.001 (0.029)	−0.032 0.080	0.019*** (0.001)	0.006 0.008	0.039*** (0.001)	0.017 0.019	0.025*** (0.000)	0.000 0.000	0.030*** (0.002)	0.039 0.047	0.005** (0.001)	0.001 0.003

BMI: body mass index; MH symp.: mental health symptomatology; NLR: neutrophil to lymphocyte ratio; SCI: systemic chronic inflammation; SES: socioeconomic status.

# Adjusted for age, sex, BMI, mental symptomatology score, overall health rating and SES.

* *p* *⩽* 0.05. **p *⩽* 0.01. ***p *⩽* 0.001.

#### Unadjusted models

Lower fruit and oily fish but higher unprocessed and processed meat intakes were associated with higher leukocyte (*F*(6, 455,522) = 591.59, *p* *⩽* 0.001, *R*^2^(adjusted) = 0.008, Cohen’s *f*^2^ = 0.008) and basophil (*F*(6, 454,673) = 55.36, *p* *⩽* 0.001, *R*^2^(adjusted) = 0.001, Cohen’s *f*^2^ = 0.0007) counts. While lower intakes of all food groups were associated with higher platelet count (*F*(6, 455,524) = 427.77, *p* *⩽* 0.001, *R*^2^(adjusted) = 0.006, Cohen’s *f*^2^ = 0.005), only lower intakes of vegetables, fruits and oily fish but higher intakes of unprocessed red and processed meat were associated with higher neutrophil count (*F*(6, 456941) = 905.19, *p* *⩽* 0.001, *R*^2^(adjusted) = 0.012, Cohen’s *f*^2^ = 0.012) and CRP levels (*F*(6, 448,637) = 273.44, *p* *⩽* 0.001, *R*^2^(adjusted) = 0.004, Cohen’s *f*^2^ = 0.004). Higher lymphocyte count, on the other hand, was associated with lower intakes of fruits and oily fish, but with higher intakes of vegetables, unprocessed red meat and non-oily fish (*F*(6, 454 673) = 15.71, *p* *⩽* 0.001, *R*^2^(adjusted) = 0.0001, Cohen’s *f*^2^ = 0.0001). Higher NLR was associated with lower intakes of vegetables, fruits, unprocessed read meat, oily and non-oily fish, but with higher amounts of processed meat (*F*(6, 448,637) = 328.31, *p* *⩽* 0.001, *R*^2^(adjusted) = 0.004, Cohen’s *f*^2^ = 0.004).

#### Adjusted models

Lower intakes of fruits, vegetables, oily and non-oily fish but higher intakes of unprocessed red and processed meat were associated with higher neutrophil counts (*F*(12, 456,941) = 1740.06, *p* *⩽* 0.001, *R*^2^(adjusted) = 0.044, Cohen’s *f*^2^ = 0.046), leukocyte counts (*F*(12, 455,522) = 1641.51, *p* *⩽* 0.001, *R*^2^(adjusted) = 0.042, Cohen’s *f*^2^ = 0.043) and CRP levels (*F*(12, 448,637) = 3190.12, *p* *⩽* 0.001, *R*^2^(adjusted) = 0.079, Cohen’s *f*^2^ = 0.085). There was also an association between lower intakes of non-oily fish and higher CRP. The same pattern of results was also observed for platelet and basophil counts; however, both non-oily fish and vegetables intakes were not associated with basophil counts (*F*(12, 454,673) = 213.73, *p* *⩽* 0.001, *R*^2^(adjusted) = 0.006, Cohen’s *f*^2^ = 0.006) and unprocessed red meat intake was not associated with platelet count (*F*(12, 455,524) = 2595.16, *p* *⩽* 0.001, *R*^2^(adjusted) = 0.064, Cohen’s *f*^2^ = 0.068). Higher lymphocyte count was associated with lower fruit and higher unprocessed red and processed meat and vegetable intakes (*F*(12, 454,673) = 402.372, *p* *⩽* 0.001, *R*^2^(adjusted) = 0.010, Cohen’s *f*^2^ = 0.010). Higher NLR was associated with lower intakes of vegetables, fruits, unprocessed red meat, oily and non-oily fish, but with higher amounts of processed meat (*F*(12, 448,637) = 684.17, *p* *⩽* 0.001, *R*^2^(adjusted) = 0.018, Cohen’s *f*^2^ = 0.018).

### SCI biomarkers predict problematic sleep

In the unadjusted model, we observed negative associations between problematic sleep index and all markers of SCI, except leukocyte count (*F*(7, 372,649) = 452.57, *p* *⩽* 0.001, *R*^2^(adjusted) = 0.008, Cohen’s *f*^2^ = 0.008). After controlling for age, sex, BMI, mental and overall health ratings and SES, leukocyte, lymphocyte and basophil counts were no longer associated with better sleep; however, lower counts of platelets, CRP and NLR and higher counts of neutrophils were associated with better sleep (*F*(13, 372,649) = 6008.846, *p* *⩽* 0.001, *R*^2^(adjusted) = 0.173, Cohen’s *f*^2^ = 0.209) (Table 3).

**Table 3.** Regression analysis summary for problematic sleep index.

	Problematic sleep index^#^		Problematic sleep index^##^	
	*β* (SE)	LL UL	*β* (SE)	LL UL
Constant	*** (0.001)	0.785 0.788	*** (0.002)	0.963 0.970
Leukocyte count	0.010 (0.001)	−0.001 0.002	−0.014 (0.001)	−0.002 0.000
Platelet count	−0.022*** (0.000)	0.000 0.000	−0.010*** (0.000)	0.000 0.000
Lymphocyte count	−0.014* (0.001)	−0.003 0.000	0.008 (0.001)	0.000 0.002
Neutrophil count	−0.042*** (0.001)	−0.004 −0.002	0.016* (0.001)	0.000 0.002
Basophil count	−0.006*** (0.004)	−0.020 −0.006	0.002 (0.001)	−0.002 0.011
C-reactive protein	−0.070*** (0.000)	−0.002 −0.002	−0.016*** (0.000)	0.000 0.000
NLR	0.008*** (0.000)	0.000 0.001	−0.005** (0.000)	−0.001 0.000
Age			−0.099*** (0.000)	−0.001 −0.001
Sex (F = 0/M = 1)			0.068*** (0.000)	0.014 0.015
BMI			−0.047*** (0.000)	−0.001 −0.001
MH symp. (higher = worse)			−0.256*** (0.000)	−0.009 −0.009
Health rating (higher: worse)			−0.220*** (0.000)	−0.033 −0.032
SES (higher : least affluent)			−0.057*** (0.000)	−0.002 −0.002

BMI: body mass index; MH symp.: mental health symptomatology; NLR: neutrophil to lymphocyte ratio; SES: socioeconomic status.

# Unadjusted.

## Adjusted for age, sex, BMI, mental health symptomatology, SES and overall health ratings.

** *p* *⩽* 0.01. ***p *⩽* 0.001.

### The role of SCI biomarkers in problematic sleep index and healthy diet score association

**Figure 1.**
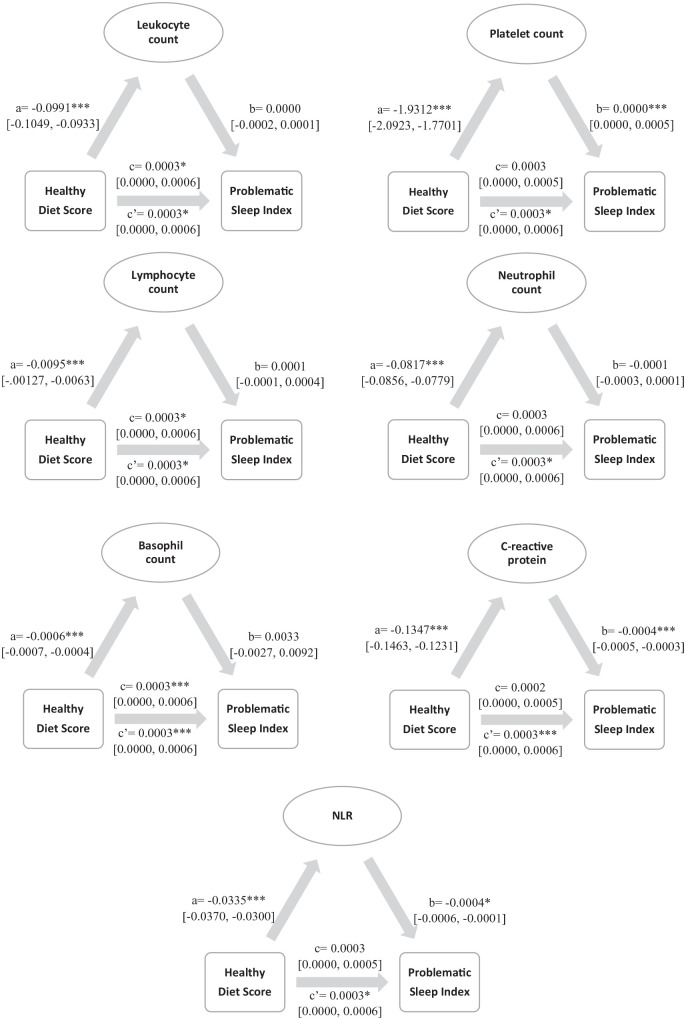
Results of the mediation analyses showing standardised coefficients, significance and confidence intervals. ***p *⩽* 0.001. *p *⩽* 0.05.

Significance and path coefficients are shown in the mediation models depicted in Figure 1, Table 4 shows indirect effects. We found significant relationships between healthy diet score and all SCI biomarkers, indicating that individuals with lower healthy diet scores had increased SCI biomarkers (paths a). We also observed (i) negative associations between problematic sleep index and neutrophil counts, CRP and NLR (showing that higher biomarker counts/levels were associated with more problematic sleep) and (ii) positive associations between problematic sleep index and platelet count (showing that counts were associated with less problematic sleep**)** (paths b). While platelet and neutrophil counts, CRP levels and NLR fully mediated the association between healthy diet score and problematic sleep index, for leukocyte, lymphocyte and basophil counts, both direct (paths c) and total (paths c′) effects of healthy diet score on problematic sleep index were significant, indicating that these SCI biomarker counts partially mediated the effects of healthy diet score on sleep problems.

**Table 4.** Standardised indirect associations between healthy diet score on problematic sleep index.

	Coefficient	SE	95% CI
Diet→Leukocyte count→Sleep	0.0000	0.0001	[−0.0001, 0.0002]
Diet→Platelet count→Sleep	0.0003	0.0001	[0.0002, 0.0005]
Diet→Lymphocyte count→Sleep	0.0000	0.0000	[0.0000, 0.0000]
Diet→Neutrophil count→Sleep	0.0001	0.0001	[−0.0001, 0.0003]
Diet→Basophil count→Sleep	0.0000	0.0000	[−0.0001, 0.0000]
Diet→C-reactive protein→Sleep	0.0006	0.0001	[0.0005, 0.0007]
Diet→NLR→Sleep	0.0001	0.0000	[0.0000, 0.0002]

CI: confidence intervals; NLR: neutrophil to lymphocyte ratio; SE: standard error.

We also examined the significance of indirect associations for each model, by using bias-corrected bootstrap confidence intervals. The confidence intervals for each indirect path were above zero for some SCI biomarkers, indicating significant indirect associations between healthy diet score and problematic sleep index through platelet and lymphocyte counts, CRP levels and NLR (see Table 4).

### Secondary analyses

Given the bidirectional nature of sleep-inflammation and diet-inflammation relationships, we have also included results from regression analyses predicting SCI from both problematic sleep index and healthy diet score (see Supplemental Results, Tables 3 to 9).

## Discussion and conclusion

To the best of our knowledge, this is the first large-scale study that aimed to investigate (1) the associations of SCI biomarkers with (1.1.) problematic sleep and (1.2.) healthy diet and (2) the role of SCI biomarkers in diet–sleep relationship. As predicted, all SCI biomarkers were negatively associated with both better sleep and healthy diet score (except platelet count for healthy diet). We found that food groups that are abundant in healthy (e.g. MED-style) diets (fruit, vegetable and oily and non-oily fish) were negatively associated with SCI markers, whereas food groups that are abundant in unhealthy (e.g. Western-style) diets (processed meat) were positively associated with SCI markers in the adjusted models. Regards to sleep, we observed negative associations with platelet counts, CRP levels (i.e. lower levels and counts were associated with better sleep) and NLR, but a positive association with neutrophil count in the adjusted model. Finally, after adjusting for age, sex, BMI, mental health symptomatology, overall health ratings and SES, platelet and neutrophil counts, CRP levels and NLR were found to be fully mediating the association between healthy diet score and problematic sleep index, whereas leukocyte, lymphocyte and basophil counts were found to be partially mediating the associations between healthy diet score and problematic sleep index. Additionally, we observed significant indirect associations between healthy diet score and problematic sleep index through platelet and lymphocyte counts, CRP levels and NLR.

Our findings showing negative associations between food groups that are abundant in healthy diets (vegetables, fruits and fish) and SCI biomarkers are consistent with the existing literature showing anti-inflammatory effects of consumption of these food groups (Esmaillzadeh et al., 2006; Imano et al., 1999; Menni et al., 2021; Nakagami et al., 2019; Wannamethee et al., 2006; Zampelas et al., 2005) and adherence MED-style diets (Bonaccio et al., 2014; Chrysohoou et al., 2004; Lopez-Garcia et al., 2004; Waldeyer et al., 2018). The beneficial effects of healthy diets have been ascribed to their high content of antioxidants, fibre and unsaturated fatty acids (Bonaccio et al., 2013); therefore, these nutrients may have contributed to this anti-inflammatory effect. Converging evidence comes from previous research showing inverse relationships between SCI and dietary antioxidant (Wisnuwardani et al., 2020), fibre (Ma et al., 2008) and polyunsaturated fatty acid (Ferrucci et al., 2006) intakes. Additionally, this negative relationship between inflammatory biomarkers and food groups that are abundant in the healthy diets could also be attributed to the healthy lifestyle associated with adherence to these diets.

On the other hand, in line with previous literature (Ali et al., 2017; Azadbakht and Esmaillzadeh, 2009; Chai et al., 2017; Ley et al., 2014), we observed positive associations with SCI biomarkers and (i) consumption of food groups that are abundant in unhealthy diets (such as processed and unprocessed red meat) and (ii) adherence to Western-style diets (Khayyatzadeh et al., 2018; Silveira et al., 2018). It is well known that unhealthy diets are characterised by higher consumption of sugar, salt, refined grains and saturated- and trans-fatty acids (Jacka, 2017; Marx et al., 2017) and, of special importance, saturated fatty acids, have been repeatedly shown to increase inflammatory markers (Mu et al., 2014; Ruiz-Núñez et al., 2016). Additionally, innate immune cells are known to (i) be affected by food intake (as nutrients can stimulate pattern recognition receptors) and (ii) start an inflammatory response to some or all of these nutrients as they do to pathogens (Hotamisligil, 2017).

In the current study, we observed associations with between better sleep and lower counts of platelets, CRP levels and NLR. Our findings extend the evidence form previous research showing negative associations with CRP levels and (i) sleep duration (Ferrie et al., 2013) and sleep quality (Lee et al., 2020; Liu et al., 2014). To the best of our knowledge, the current study is the first one to show associations of poor sleep quality (i.e. an index of sleep quality beyond mere duration) with (i) platelet counts and (ii) NLR in generally healthy population by using self-reported sleep measures. Our findings show that poor sleep may induce SCI. Alternatively, given that increased counts of platelets, CRP levels and NLR are associated with SCI, an increase in these pro-inflammatory signals may have affected various sleep outcomes via neural, humoral, blood–brain barrier transport and/or cellular mechanisms (for details of possible mechanisms of action, see Bryant et al., 2004; Irwin, 2019) and therefore predicted more problematic sleep.

Unlike previous studies showing negative associations between leukocyte counts and subjective (Nishitani and Sakakibara, 2007) and objective (Obayashi et al., 2016) sleep quality, in the current study, we did not observe this association. It is important to note that half of the participants recruited by Nishitani and Sakakibara (2007) were involved in shift working practices. Given the association between shift working and SCI (Puttonen et al., 2011), their findings may partly be attributed to the higher counts of leukocytes observed in the shift workers skewing findings towards significance. Additionally, Obayashi and colleagues (2016) used actigraphy to measure sleep quality. It is well known that there is a poor agreement between sleep questions typically used to assess sleep quality in epidemiologic studies and actigraphy-derived sleep quality (Girschik et al., 2012); hence, self-report questions in the UKB study might not be sensitive enough to capture the association between sleep quality and leukocyte count.

Interestingly, although intervention studies found that long-term sleep restriction (5 days of restricted/shortened sleep, 4 h/night: 03.00–07.00 h) increased neutrophil counts (Lasselin et al., 2015), we observed a positive association between better sleep and neutrophil counts. Given that (i) the NLR is a robust biomarker of SCI (Zahorec, 2021) and (ii) we observed a negative association between NLR and problematic sleep index, we could conclude that neutrophil counts alone might not be a sensitive marker for predicting habitual sleep outcomes, but they may be a useful indicator of altered SCI as a result of chronic sleep loss and/or sleep disorders.

Although it has been shown that both (i) diet quality (Hepsomali and Groeger, 2021) and (ii) reduced dietary inflammation index scores (Godos et al., 2019) were associated with better sleep quality, the current study was the first to investigate and show the mediating role of various inflammatory markers on diet and sleep quality relationship in a large generally healthy population. These findings not only link the existing evidence showing negative associations between (i) healthy diets and inflammatory markers (Bonaccio et al., 2014; Chrysohoou et al., 2004; Lopez-Garcia et al., 2004; Waldeyer et al., 2018) and (ii) inflammatory markers and sleep quality (Lee et al., 2020; Liu et al., 2014; Nishitani and Sakakibara, 2007; Obayashi et al., 2016), but also add to the wealth of evidence showing the mediating role of SCI on the relationship between diet and chronic diseases (Soory, 2012). Moreover, directly affecting metabolic and immunologic responses, diet could also influence sleep outcomes indirectly via influencing the gut microbiota (and vice versa), which is known to play a role in driving SCI (Hakansson and Molin, 2011). In fact, a recent review has proposed a link between the gut microbiota and circadian rhythms, potentially contributing to poor sleep and/or sleep disorders (Teichman et al., 2020). Therefore, adherence to healthy diets might lead to an anti-inflammatory response as a result of changes in the gut microbiota’s composition and diversity and may result in positive sleep outcomes.

There are several notable limitations in the current research. First of all, although our study benefits from a large population-based sample and a wide range of covariates available for adjustment, including mental health, which is known to have a bidirectional relationship between both sleep outcomes (Breslau et al., 1996; Gillin et al., 1979; Neckelmann et al., 2007; Soehner and Harvey, 2012) and SCI (Cryan and Dinan, 2012; Phillips et al., 2018), due to the cross-sectional nature of the study, we could not determine causal relationships. This is especially important because while some researchers criticised conducting mediation analyses using cross-sectional data (Fairchild and McDaniel, 2017), others posited that atemporal mediation (as in the current study) can still be demonstrated without inferring causality (Hayes, 2017; Hayes and Rockwood, 2017; MacKinnon et al., 2007). Secondly, the UKB data set includes self-report sleep measures, and as such the possibility of potential bias and/or measurement errors should not be ruled out. Thirdly, although we observed small effect sizes in our study, it is well known that effect sizes observed in large cohort studies are substantially smaller compared to case–control/clinical studies. Sample characteristics of case–control/clinical studies (i.e. extreme cases and/or use of medication inflating the effect sizes) and/or reduced sensitivity of UKB measures compared to the ones utilised in case–control/clinical studies may partly account for the small effect sizes observed here. However, it is accepted that even though small to modest overall effect sizes may be of limited clinical relevance, such differences may have substantial consequences for whole populations. Fourthly, associations we observed are based on single measurements of SCI biomarkers; however, although intraindividual variabilities of inflammatory biomarkers have been observed before (e.g. deGoma et al., 2012), single measurements of these inflammatory biomarkers have been shown to predict a range of health outcomes (e.g. Chung et al., 2005; Ruggiero et al., 2007; Wium-Andersen et al., 2013). Fifthly, the time of the blood draw is unknown in the current study, hence results should be interpreted with caution as some SCI biomarkers show circadian rhythms (e.g. Lange et al., 2022). Finally, as we adjusted our models for a number of covariates (but not for smoking, physical activity, medication, sleep disorders etc.), our findings are potentially sensitive to selection bias or reduced power, arising from missing data. Further interventional studies that examine the temporal order of the associations are warranted to replicate our results, preferably by using objective measures and multiple biomarker collections, and while controlling for chronic diseases, and ultimately develop a better understanding of the mechanisms underlying the interplay between diet, SCI and sleep.

In conclusion, we found negative associations between SCI biomarkers and habitual consumption of food groups (vegetables, fruits and seafood) that are abundant in healthy diets. In contrast, positive associations were observed between SCI biomarkers and habitual consumption of food groups (processed meat) that are abundant in unhealthy diets. Although a reduction in some SCI biomarkers (platelets, CRP and NLR) predicted better sleep quality, neutrophil counts, CRP levels and NLR fully; and leukocyte, lymphocyte and basophil counts partially mediated diet and sleep relationship. It is clear from our findings that healthy diets are associated with lower SCI, and lower SCI biomarkers predict better sleep quality, and therefore, adherence to a good quality diet may represent a promising therapeutic, preventative and/or self-management strategy for sleep disorders/issues and poor sleep-related long-term health outcomes.

## Supplemental Material

sj-docx-1-jop-10.1177_02698811221112932 – Supplemental material for Examining the role of systemic chronic inflammation in diet and sleep relationship Click here for additional data file. Supplemental material, sj-docx-1-jop-10.1177_02698811221112932 for Examining the role of systemic chronic inflammation in diet and sleep relationship by Piril Hepsomali and John A Groeger in Journal of Psychopharmacology

sj-docx-2-jop-10.1177_02698811221112932 – Supplemental material for Examining the role of systemic chronic inflammation in diet and sleep relationship Click here for additional data file. Supplemental material, sj-docx-2-jop-10.1177_02698811221112932 for Examining the role of systemic chronic inflammation in diet and sleep relationship by Piril Hepsomali and John A Groeger in Journal of Psychopharmacology

sj-docx-3-jop-10.1177_02698811221112932 – Supplemental material for Examining the role of systemic chronic inflammation in diet and sleep relationship Click here for additional data file. Supplemental material, sj-docx-3-jop-10.1177_02698811221112932 for Examining the role of systemic chronic inflammation in diet and sleep relationship by Piril Hepsomali and John A Groeger in Journal of Psychopharmacology
