# Supplementary material for: Examining the role of systemic chronic inflammation in diet and sleep relationship
Source: J Psychopharmacol. 2022 Jul 21;36(9):1077–86. doi: 10.1177/02698811221112932 (PMC9516605; doi:10.1177/02698811221112932)
Supplement: sj-docx-1-jop-10.1177_02698811221112932 – Supplemental material for Examining the role of systemic chronic inflammation in diet and sleep relationship [file sj-docx-1-jop-10.1177_02698811221112932.docx]

**Examining the role of systemic chronic inflammation in diet and sleep relationship**

Piril Hepsomali^1,2*^& John A. Groeger^3^

^1^ University of Roehampton, School of Psychology, London, United Kingdom

^2^ Unilever R&D, Colworth Science Park, Bedford, United Kingdom

^3^ Nottingham Trent University, School of Social Sciences, Department of Psychology, Nottingham, United Kingdom

^*^ Correspondence concerning this article should be addressed to Piril Hepsomali, University of Roehampton, School of Psychology, London, United Kingdom, email: [P.Hepsomali@roehampton.ac.uk](mailto:P.Hepsomali@roehampton.ac.uk)

**Acknowledgements:** This research has been conducted using the UK Biobank Resource under Application Number ‘61818’. PH was affiliated with Unilever UK Central Resources Limited at the time of data analysis and is currently affiliated with University of Roehampton.

**Data availability:** All relevant data are within the paper and its supplementary material.

**Author contributions:** PH analysed the data and wrote the manuscript with input from JG, who also contributed to the revision of the manuscript critically for important intellectual content. Both PH and JAG approved the submitted version.

**Funding:** Funds received from Unilever UK Central Resources Limited to cover application fees.

**Conflicts of interest:** PH was employed by Unilever UK Central Resources Limited until May 2021. JAG has received research funding, consultancy, travel support, and speaking fees from various industrial companies of which Precision Biotics was the only biotechnology company that develops and commercialises vitamins/supplements.
