# Supplementary material for: Examining the role of systemic chronic inflammation in diet and sleep relationship
Source: J Psychopharmacol. 2022 Jul 21;36(9):1077–86. doi: 10.1177/02698811221112932 (PMC9516605; doi:10.1177/02698811221112932)
Supplement: sj-docx-2-jop-10.1177_02698811221112932 – Supplemental material for Examining the role of systemic chronic inflammation in diet and sleep relationship [file sj-docx-2-jop-10.1177_02698811221112932.docx]

**Supplementary Methods**

**Recruitment procedure:** UK Biobank issued mailed invitations to about 9 million persons aged 40–69 years living within 25 miles (40.23 km) from the 21 study assessment centres (generally in big cities) and registered with the National Health Service in the United Kingdom. More than 500,000 participants responded to this invitation (response rate 5.5 %). Although individuals with acute and chronic diseases were not excluded, given the low response rate, the sample consists of generally health middle aged adults.

**Assessment of diet:** In order to assess diet, we used UKB’s touchscreen questionnaire on food frequency at baseline. Data fields of interest can be seen below.

| **Variable** | **Question** | **Field ID** |
| --- | --- | --- |
| Cooked vegetable intake | On average how many heaped tablespoons of cooked vegetables would you eat per day? | 1289 |
| Salad/raw vegetable intake | On average how many heaped tablespoons of raw vegetables would you eat per day? | 1299 |
| Fresh fruit intake | About how many pieces of fresh fruit would you eat per day? | 1309 |
| Dried fruit intake | About how many pieces of dried fruit would you eat per day? | 1319 |
| Oily fish intake | How often do you eat oily fish? (e.g., sardines, salmon, mackerel, herring); coded as never, less than once a week, once a week, 2-4 times a week, 5-6 times a week, once or more daily, do not know, and prefer not to answer | 1329 |
| Non-oily fish intake | How often do you eat other types of fish? (e.g., cod, tinned tuna, haddock); coded as never, less than once a week, once a week, 2-4 times a week, 5-6 times a week, once or more daily, do not know, and prefer not to answer | 1339 |
| Processed meat intake | How often do you eat processed meats (such as bacon, ham, sausages, meat pies, kebabs, burgers, chicken nuggets)?; coded as never, less than once a week, once a week, 2-4 times a week, 5-6 times a week, once or more daily, do not know, and prefer not to answer | 1349 |
| Poultry intake | How often do you eat chicken, turkey or other poultry? (Do not count processed meats); coded as never, less than once a week, once a week, 2-4 times a week, 5-6 times a week, once or more daily, do not know, and prefer not to answer | 1359 |
| Beef intake | How often do you eat beef? (Do not count processed meats); coded as never, less than once a week, once a week, 2-4 times a week, 5-6 times a week, once or more daily, do not know, and prefer not to answer | 1369 |
| Lamb/mutton intake | How often do you eat lamb/mutton? (Do not  count processed meats); coded as never, less than once a week, once a week, 2-4 times a week, 5-6 times a week, once or more daily, do not know, and prefer not to answer | 1379 |
| Pork intake | How often do you eat pork? (Do not count processed meats); coded as never, less than once a week, once a week, 2-4 times a week, 5-6 times a week, once or more daily, do not know, and prefer not to answer) | 1389 |
| Bread intake | How many slices of bread do you eat each week? | 1438 |
| Bread intake | What type of bread do you mainly eat?; coded as white, brown, wholemeal or wholegrain, other type of bread, do not know, prefer not to answer | 1448 |
| Cereal intake | How many bowls of cereal do you eat a week? | 1458 |
| Cereal intake | What type of cereal do you mainly eat?; coded as brain, biscuit, oat, muesli, other, do not know, prefer not to answer | 1468 |

In order to calculate healthy diet score we utilised Bradbury et al. (2018)’s estimations that was based on consumption of commonly eaten food groups following recommendations on dietary priorities for cardiometabolic health (Fruits: ≥ 3 servings/day, Vegetables: ≥ 3 servings/day, Fish: ≥ 2 servings/week, Processed meats: ≤ 1 serving/week, Unprocessed red meats: ≤ 1.5 servings/week, Whole grains: ≥ 3servings/day, Refined grains: ≤ 1.5 servings/day). 1 point was given for adherence to each recommendation and all scores were added where higher scores represented higher health diet scores. In order to calculate total vegetable and fruit intake, we summed cooked and salad/raw vegetable intake and fresh and dried fruit intake, respectively. We also added beef, lamb/mutton, and pork intake scores and oily and non-oily fish intake scores to create total unprocessed red meat intake and total fish intake scores, respectively.

**Assessment of sleep:** In order to assess sleep, we used UKB’s touchscreen questionnaire on sleep at baseline. Data fields of interest can be seen below.

| **Variable** | **Question** | **Field ID** |
| --- | --- | --- |
| Sleep duration | About how many hours sleep do you get in every 24 hours? (please include naps) | 1160 |
| Chronotype | Do you consider yourself to be?; coded as definitely a morning person, more a morning than evening person, more an evening than a morning person, definitely an evening person, do not know, prefer not to answer | 1180 |
| Sleeplessness/insomnia | Do you have trouble falling asleep at night or do you wake up in the middle of the night?; coded as never/rarely, sometimes, usually, prefer not to answer | 1200 |
| Snoring | Does your partner or a close relative or friend complain about your snoring?; coded as yes, no, do not know, prefer not to answer | 1210 |
| Daytime dozing / sleeping | How likely are you to doze off or fall asleep during the daytime when you don't mean to? (e.g., when working, reading, or driving) | 1220 |
| Nap during day | Do you have a nap during the day?; coded as never/rarely, sometimes, usually, prefer not to answer | 1190 |
| Getting up in morning | On an average day, how easy do you find getting up in the morning?; coded as not at all easy, not very easy, fairly easy, very easy, do not know, prefer not to answer | 1170 |

In order to calculate Problematic Sleep Index (Groeger & Hepsomali, under review) first we recoded (1) sleep duration into five categories (much lower than recommended, lower than recommended, recommended, higher than recommended, much higher than recommended) based on age-specific recommended daily sleep durations (Hirshkowitz et al., 2015). Second, we created a new (2) daytime sleepiness score by combining the responses for the questions about dozing and napping by calculating a single factor score using Principal Components Analysis (PCA). Third, we calculated a new (3) ease of waking score by regressing out any potential contribution of chronotype from getting up in morning. The new scores of (2) and (3), the recoded sleep duration (1) and the original (4) sleeplessness/insomnia and (5) snoring data were combined into a single factor using PCA and rescaled such that that ‘1’ represented the best possible sleep, that is sleeping for the recommended duration, waking easily following that sleep, not sleeping during the day, falling asleep easily and or not waking, or snoring, during sleep. Better sleep quality scores obtained by our index are associated with (i) lower numbers of mental health symptomatology reported (*r*(*410134*)=-0.314, *p*<0.001) and (ii) better overall health ratings (*r*(*408975*)=-0.309, *p*<0.001).

**Assessment of mental health:** For mental health assessment, we utilised UKB’s touchscreen questionnaire on psychological factors and mental health at baseline. Data fields of interest can be seen below.

| **Variable** | **Question*** | **Field ID** |
| --- | --- | --- |
| Mood swings | Does your mood often go up and down? | 1920 |
| Miserableness | Do you ever feel 'just miserable' for no reason? | 1930 |
| Irritability | Are you an irritable person? | 1940 |
| Sensitivity/hurt feelings | Are your feelings easily hurt ? | 1950 |
| Fed-up feelings | Do you often feel 'fed-up'? | 1960 |
| Nervous feelings | Would you call yourself a nervous person? | 1970 |
| Worrier/anxious feelings | Are you a worrier? | 1980 |
| Tense/highly strung | Would you call yourself tense or 'highly strung'? | 1990 |
| Worry too long after embarrassment | Do you worry too long after an embarrassing experience? | 2000 |
| Suffer from nerves | Do you suffer from 'nerves'? | 2010 |
| Loneliness/isolation | Do you often feel lonely? | 2020 |
| Guilty feelings | Are you often troubled by feelings of guilt? | 2030 |
| Risk taking | Would you describe yourself as someone who takes risks? | 2040 |

* All coded as ‘yes’, ‘no’, ‘do not know’, ‘prefer not to answer’.

As in our previous study (Hepsomali & Groeger, 2021), in order to create total mental health symptomatology score, we calculated the total mental health complaints reported by adding up participant’s answers to the questions described above, where higher numbers represent more mental health-related symptomatology.

**Other variables**

*Overall Health Rating.* Responses to a self-report question of "In general how would you rate your overall health?" (Field ID:2178) was used as a covariate (coded as excellent, good, fair, poor, do not know, prefer not to answer)

*Deprivation Index.* Townsend deprivation index (a measure of material deprivation) at recruitment (Field ID: 189) was used as a socioeconomic status covariate.

**References**

Bradbury, K. E., Young, H. J., Guo, W., & Key, T. J. (2018). Dietary assessment in UK Biobank: an evaluation of the performance of the touchscreen dietary questionnaire. *Journal of Nutritional Science*, *7*, e6, Article e6. <https://doi.org/10.1017/jns.2017.66>

Groeger, J. A., & Hepsomali, P. (under review). *The Inequality of Sleep Quality: Social Deprivation and Ethnicity Affect the Sleep Quality of Middle Aged and Older Adults*.

Hepsomali, P., & Groeger, J. A. (2021). Diet, sleep, and mental health: Insights from the UK Biobank study. *Nutrients*, *13*(8), 2573. <https://doi.org/https://doi.org/10.3390/nu13082573>

Hirshkowitz, M., Whiton, K., Albert, S. M., Alessi, C., Bruni, O., DonCarlos, L., . . . Adams Hillard, P. J. (2015). National Sleep Foundation's sleep time duration recommendations: methodology and results summary. *Sleep Health*, *1*(1), 40-43. <https://doi.org/10.1016/j.sleh.2014.12.010>
