# Supplementary material for: Examining the role of systemic chronic inflammation in diet and sleep relationship
Source: J Psychopharmacol. 2022 Jul 21;36(9):1077–86. doi: 10.1177/02698811221112932 (PMC9516605; doi:10.1177/02698811221112932)
Supplement: sj-docx-3-jop-10.1177_02698811221112932 – Supplemental material for Examining the role of systemic chronic inflammation in diet and sleep relationship [file sj-docx-3-jop-10.1177_02698811221112932.docx]

**Supplementary Results**

**Table 1.** Baseline characteristics of 449,084 participants according to SCI biomarkers*

|  | *N* | **Leukocyte count**  (10^9 cells/L) | **Platelet count**  (10^9 cells/L) | **Lymphocyte count**  (10^9 cells/L) | **Neutrophil count**  (10^9 cells/L) | **Basophil count**  (10^9 cells/L) | **C-reactive protein**  (mg/L) | **NLR** |
| --- | --- | --- | --- | --- | --- | --- | --- | --- |
|  |  | *(25^th^,* ***50 ^th^****, 75^th^ p.)* | *(25^th^,* ***50 ^th^****, 75^th^ p.)* | *(25^th^,* ***50 ^th^****, 75^th^ p.)* | *(25^th^,* ***50 ^th^****, 75^th^ p.)* | *(25^th^,* ***50 ^th^****, 75^th^ p.)* | *(25^th^,* ***50 ^th^****, 75^th^ p.)* |  |
| **Sex**  Female  Male | 243432  205652 | 5.60,**6.61**, 7.81  5.65,**6.67**, 7.87 | 226.00, **261.00**, 300.40  201.80, **233.70**, 269.00 | 1.58, **1.91**, 2.34  1.48, **1.80**, 2.20 | 3.23, **4.00**, 4.91  3.30, **4.06**, 5.00 | 0.00, **0.02**, 0.04  0.00, **0.02**, 0.04 | 0.65, **1.37**, 2.96  0.66, **1.28**, 2.53 | 1.61, **2.06**, 2.65  1.73, **2.23**, 2,91 |
| **Age**  40-44  45-49  50-54  55-59  60-64  65+ | 46280  59216  68498  81663  109409  84018 | 5.60, **6.64**, 7.90  5.58, **6.60**, 7.90  5.54, **6.56**, 7.76  5.55, **6.54**, 7.70  5.68, **6.66,** 7.82  5.80, **6.80**, 7.99 | 216.90, **251.40**, 291.00  218.00, **253.00**, 292.10  218.00, **252.45**, 292.00  215.00, **249.50**, 288.00  211.50, **245.90**, 284.40  206.20, **241.00**, 280.00 | 1.51, **1.87**, 2.26  1.50, **1.86**, 2.26  1.52, **1.90**, 2.30  1.52, **1.90**, 2.30  1.51, **1.89**, 2.30  1.50, **1.85**, 2.28 | 3.21, **4.05**, 5.05  3.21, **4.03**, 5.02  3.20, **3.95**, 4.90  3.20, **3.90**, 4.80  3.30, **4.00**, 4.90  3.40, **4.18**, 5.10 | 0.00, **0.02**, 0.04  0.00, **0.02**, 0.04  0.00, **0.02**, 0.04  0.00, **0.02**, 0.04  0.00, **0.02**, 0.04  0.00, **0.02**, 0.04 | 0.50, **1.03**, 2.26  0.54, **1.10**, 2.37  0.61, **1.23**, 2.64  0.65, **1.32**, 2.74  0.73, **1.44**, 2.92  0.82, **1.58**, 3.11 | 1.68, **2.15,** 2.75  1.69, **2.16**, 2.78  1.62, **2.09**, 2.69  1.61, **2.06**, 2.66  1.65, **2.12**, 2.75  1.73, **2.23**, 2.92 |
| **BMI**  <18.5 (Underweight)  18.5-25 (Normal)  25-30 (Overweight)  30+ (Obese) | 2283  146778  191318  108705 | 5.11, **6.22**, 7.61  5.36, **6.30**, 7.49  5.65, **6.61**, 7.78  6.10, **7.13**, 8.39 | 209.80, **245.60**, 287.00  213.90, **248.00**, 286.50  212.80, **246.80**, 285.10  214.60, **251.00**, 291.30 | 1.30, **1.66**, 2.09  1.44, **1.79**, 2.17  1.51, **1.88**, 2.29  1.64, **2.00**, 2.45 | 3.00, **3.84**, 5.00  3.10, **3.84**, 4.76  3.27, **4.00**, 4.90  3.50, **4.30**, 5.29 | 0.00, **0.02**, 0.05  0.00, **0.02**, 0.04  0.00, **0.02**, 0.04  0.00, **0.02**, 0.05 | 0.24, **0.43**, 1.02  0.41, **0.77**, 1.56  0.73, **1.35**, 2.55  1.37, **2.54**, 4.78 | 1.76, **2.33**, 3.11  1.66, **2.15**, 2.80  1.65, **2.12,** 2.75  1.66, **2.13**, 2.75 |
| **Overall Health Rating**  Excellent  Good  Fair  Poor | 74469  260913  93940  19762 | 5.39, **6.30**, 7.37  5.60, **6.59**, 7.71  5.90, **7.00**, 8.30  6.20, **7.43**, 8.93 | 213.00, **246.00**, 283.00  213.80, **248.00**, 286.20  213.50, **250.00**, 291.00  213.00, **252.80**, 297.40 | 1.50, **1.80**, 2.20  1.51, **1.87**, 2.28  1.55, **1.91**, 2.39  1.51, **1.95**, 2.45 | 3.09, **3.79**, 4.60  3.22, **3.99**, 4.87  3.47, **4.30**, 5.30  3.68, **4.61**, 5.81 | 0.00, **0.02**, 0.04  0.00, **0.02**, 0.04  0.00, **0.02**, 0.05  0.00, **0.03**, 0.06 | 0.49, **0.92**, 1.83  0.64, **1.26**, 2.53  0.89, **1.84**, 3.76  1.19, **2.57**, 5.63 | 1.62, **2.07,** 2.66  1.65, **2.11**, 2.72  1.71, **2.21**, 2.88  1.78, **2.35**, 3.18 |
| **MH symp.**  1 (Low)  2  3  4  5 (High)  **SES**  1 (Most affluent)  2  3  4  5 (Least affluent) | 113637  113254  52480  127473  94393  100359  100385  100377  100376  100373 | 5.60, **6.60**, 7.78  5.60, **6.60**, 7.80  5.60, **6.62**, 7.84  5.63, **6.67**, 7.88  5.70, **6.73**, 7.99  5.60, **6.52**, 7.66  5.60, **6.58**, 7.70  5.60, **6.60**, 7.80  5.66, **6.70**, 7.90  5.73, **6.87**, 8.20 | 210.00, **243.60**, 282.00  212.00, **247.00**, 285.60  213.70, **248.10**, 287.60  215.00, **250.00**, 289.10  217.00, **252.30**, 292.00  213.70, **247.80**, 285.80  213.00, **247.30**, 286.00  213.90, **248.00**, 287.00  213.00, **248.00**, 287.00  213.50, **249.60**, 290.00 | 1.50, **1.86**, 2.27  1.50, **1.87**, 2.29  1.51, **1.88**, 2.30  1.51, **1.89**, 2.30  1.52, **1.90**, 2.30  1.50, **1.84**, 2.23  1.50, **1.85**, 2.25  1.50, **1.87**, 2.27  1.51, **1.89**, 2.30  1.55, **1.91**, 2.38 | 3.23, **4.00**, 4.90  3.24, **4.00**, 4.90  3.25, **4.00**, 4.96  3.28, **4.03**, 4.99  3.30**, 4.10**, 5.07  3.21, **3.95**, 4.81  3.23, **3.99**, 4.88  3.26, **4.00**, 4.90  3.29, **4.06**, 5.00  3.30, **4.16**, 5.20 | .00, .**02**, .04  .00, .**02**, .04  .00, .**02**, .04  .00, .**02**, .04  .00, .**02**, .04  .00, .**02**, .04  .00, .**02**, .04  .00, .**02**, .04  .00, .**02**, .04  .00, .**02**, .05 | .65, **1.29** , 2.63  .65, **1.30** , 2.67  .65, **1.32**, 2.74  .66, **1.34**, 2.82  .67, **1.40** , 2.98  0.62, **1.22**, 2.46  0.64, **1.26**, 2.55  0.64, **1.30**, 2.68  0.66, **1.35**, 2.86  0.73, **1.56**, 3.34 | 1.66, **2.13**, 2.76  1.65, **2.12**, 2.76  1.66, **2.13**, 2.76  1.66, **2.13**, 2.76  1.67, **2.15**, 2.78  1.66, **2.13**, 2.74  1.66, **2.13**, 2.75  1.66, **2.13**, 2.75  1.66, **2.14**, 2.78  1.65, **2.15**, 2.81 |

MH symp.: mental health symptomatology; NLR: Neutrophil to Leukocyte ratio; SES: socioeconomic status

*As SCI biomarkers were not normally distributed, median and quartiles were reported.

**Table 2.** Correlation matrix showing the relationships between problematic sleep index, healthy diet score, and SCI biomarkers in two halves of the dataset (half one)

|  |  |  | **1** | **2** | **3** | **4** | **5** | **6** | **7** | **8** | **NLR** |
| --- | --- | --- | --- | --- | --- | --- | --- | --- | --- | --- | --- |
| **Half one** | **1. Problematic Sleep Index** | *r* | 1 | .015*** | -.052*** | -.037*** | -.021*** | -.057*** | -.018*** | -.082*** | -.023*** |
|  |  | *N* |  | 204634 | 196347 | 196350 | 196006 | 196006 | 196006 | 192458 | 196001 |
|  | **2. Healthy Diet Score** | *r* |  | 1 | -.070*** | 0.001 | -.006*** | -.087** | -.012*** | -.052*** | -.051*** |
|  |  | *N* |  |  | 238374 | 238375 | 237958 | 237958 | 237958 | 233619 | 237953 |
|  | **3. Leukocyte count** | *r* |  |  | 1 | .202*** | .719*** | .786*** | .267*** | .182*** | .199*** |
|  |  | *N* |  |  |  | 238371 | 237958 | 237958 | 237958 | 227549 | 237953 |
|  | **4. Platelet count** | *r* |  |  |  | 1 | .072*** | .226*** | .077*** | .123*** | .034*** |
|  |  | *N* |  |  |  |  | 237955 | 237955 | 237955 | 227550 | 237950 |
|  | **5. Lymphocyte count** | *r* |  |  |  |  | 1 | .164*** | .206*** | .021*** | -.276*** |
|  |  | *N* |  |  |  |  |  | 237958 | 237958 | 227162 | 237953 |
|  | **6. Neutrophil count** | *r* |  |  |  |  |  | 1 | .155*** | .237*** | .540*** |
|  |  | *N* |  |  |  |  |  |  | 237958 | 227162 | 237953 |
|  | **7. Basophil count** | *r* |  |  |  |  |  |  | 1 | .050*** | -.019*** |
|  |  | *N* |  |  |  |  |  |  |  | 227162 | 237953 |
|  | **8. C-reactive protein** | *r* |  |  |  |  |  |  |  | 1 | .156*** |
|  |  | *N* |  |  |  |  |  |  |  |  | 227157 |
|  | **9. NLR** | *r* |  |  |  |  |  |  |  |  | 1 |
|  |  | *N* |  |  |  |  |  |  |  |  |  |

NLR: Neutrophil to Leukocyte ratio

**Table 2 (cont).** Correlation matrix showing the relationships between problematic sleep index, healthy diet score, and SCI biomarkers in two halves of the dataset (half two)

|  |  |  | **1** | **2** | **3** | **4** | **5** | **6** | **7** | **8** | **NLR** |
| --- | --- | --- | --- | --- | --- | --- | --- | --- | --- | --- | --- |
| **Half two** | **1. Problematic Sleep Index** | *r* | 1 | .017*** | -.053*** | -.041*** | -.021*** | -.055*** | -.018*** | -.081*** | -.020*** |
|  |  | *N* |  | 205583 | 197531 | 197531 | 197147 | 197147 | 197147 | 193596 | 197147 |
|  | **2. Healthy Diet Score** | *r* |  | 1 | -.074*** | 0.003 | -.007*** | -.086** | -.010*** | -.049*** | -.048*** |
|  |  | *N* |  |  | 239775 | 239776 | 239307 | 239307 | 239307 | 234931 | 239305 |
|  | **3. Leukocyte count** | *r* |  |  | 1 | .209*** | .691*** | .803*** | .232*** | .196*** | .210*** |
|  |  | *N* |  |  |  | 239774 | 239307 | 239307 | 239307 | 228971 | 239305 |
|  | **4. Platelet count** | *r* |  |  |  | 1 | .072*** | .226*** | .073*** | .125*** | .038*** |
|  |  | *N* |  |  |  |  | 239306 | 239306 | 239306 | 228972 | 239304 |
|  | **5. Lymphocyte count** | *r* |  |  |  |  | 1 | .144*** | .162*** | .021*** | -.273*** |
|  |  | *N* |  |  |  |  |  | 239307 | 239307 | 228528 | 239305 |
|  | **6. Neutrophil count** | *r* |  |  |  |  |  | 1 | .143*** | .242*** | .531*** |
|  |  | *N* |  |  |  |  |  |  | 239307 | 228528 | 239305 |
|  | **7. Basophil count** | *r* |  |  |  |  |  |  | 1 | .054*** | -.017*** |
|  |  | *N* |  |  |  |  |  |  |  | 228528 | 239305 |
|  | **8. C-reactive protein** | *r* |  |  |  |  |  |  |  | 1 | .164*** |
|  |  | *N* |  |  |  |  |  |  |  |  | 228526 |
|  | **9. NLR** | *r* |  |  |  |  |  |  |  |  | 1 |
|  |  | *N* |  |  |  |  |  |  |  |  |  |

NLR: Neutrophil to Leukocyte ratio

**Table 3**. Regression analysis summary for leukocyte count

| Model | | Unstandardized Coefficients | | Standardized Coefficients | t | Sig. | 95.0% Confidence Interval for B | |
| --- | --- | --- | --- | --- | --- | --- | --- | --- |
|  |  | B | Std. Error | Beta |  |  | Lower Bound | Upper Bound |
| 1 | (Constant) | 7.854 | .025 |  | 320.004 | .000 | 7.806 | 7.902 |
|  | Problematic Sleep Index (higher=better sleep) | -.987 | .031 | -.050 | -31.461 | .000 | -1.048 | -.925 |
|  | Healthy Diet Score  (higher=better diet) | -.130 | .003 | -.071 | -44.755 | .000 | -.136 | -.124 |
| 2 | (Constant) | 4.699 | .045 |  | 104.045 | .000 | 4.610 | 4.788 |
|  | Problematic Sleep Index (higher=better sleep) | -.010 | .034 | -.001 | -.307 | .759 | -.077 | .056 |
|  | Healthy Diet Score  (higher=better diet) | -.099 | .003 | -.054 | -33.662 | .000 | -.105 | -.093 |
|  | Age | .006 | .000 | .024 | 15.163 | .000 | .006 | .007 |
|  | Sex (F=0/M=1) | -.051 | .007 | -.012 | -7.422 | .000 | -.065 | -.038 |
|  | BMI | .053 | .001 | .119 | 72.077 | .000 | .052 | .054 |
|  | MH Symp. (higher=worse) | -.005 | .001 | -.007 | -4.095 | .000 | -.007 | -.002 |
|  | Health Rating (higher=worse) | .299 | .005 | .102 | 58.113 | .000 | .289 | .309 |
|  | SES  (higher=least affluent) | .027 | .001 | .039 | 24.240 | .000 | .025 | .029 |

*Model 1: F*(2, 390832)= 1519.026, *p≤.*001, *R^2^*(adjusted) = 0.008, *Cohen’s f ^2^*= 0.008

*Model 2: F*(8, 390832)= 2021.492, *p≤.*001, *R^2^*(adjusted) = 0.040, *Cohen’s f ^2^*= 0.041

**Table 4**. Regression analysis summary for platelet count

| Model | | Unstandardized Coefficients | | Standardized Coefficients | t | Sig. | 95.0% Confidence Interval for B | |
| --- | --- | --- | --- | --- | --- | --- | --- | --- |
|  |  | B | Std. Error | Beta |  |  | Lower Bound | Upper Bound |
| 1 | (Constant) | 269.328 | .695 |  | 387.408 | .000 | 267.966 | 270.691 |
|  | Problematic Sleep Index (higher=better sleep) | -21.610 | .889 | -.039 | -24.321 | .000 | -23.351 | -19.868 |
|  | Healthy Diet Score  (higher=better diet) | .117 | .082 | .002 | 1.418 | .156 | -.045 | .278 |
| 2 | (Constant) | 282.552 | 1.261 |  | 224.056 | .000 | 280.080 | 285.023 |
|  | Problematic Sleep Index (higher=better sleep) | -5.869 | .948 | -.011 | -6.193 | .000 | -7.726 | -4.012 |
|  | Healthy Diet Score  (higher=better diet) | -1.930 | .082 | -.038 | -23.476 | .000 | -2.091 | -1.768 |
|  | Age | -.453 | .012 | -.061 | -38.712 | .000 | -.476 | -.430 |
|  | Sex (F=0/M=1) | -28.714 | .193 | -.239 | -148.590 | .000 | -29.093 | -28.335 |
|  | BMI | .451 | .021 | .036 | 21.977 | .000 | .411 | .491 |
|  | MH Symp. (higher=worse) | .012 | .031 | .001 | .379 | .705 | -.050 | .074 |
|  | Health Rating (higher=worse) | 2.199 | .143 | .027 | 15.328 | .000 | 1.918 | 2.480 |
|  | SES  (higher=least affluent) | .046 | .031 | .002 | 1.478 | .139 | -.015 | .108 |

*Model 1: F*(2, 390837)= 296.29, *p≤.*001, *R^2^*(adjusted) = 0.002, *Cohen’s f ^2^*= 0.002

*Model 2: F*(8, 390837)= 3140.15, *p≤.*001, *R^2^*(adjusted) = 0.061, *Cohen’s f ^2^*= 0.064

**Table 5**. Regression analysis summary for lymphocyte count

| Model | | Unstandardized Coefficients | | | Standardized Coefficients | t | Sig. | 95.0% Confidence Interval for B | |
| --- | --- | --- | --- | --- | --- | --- | --- | --- | --- |
|  |  | B | | Std. Error | Beta |  |  | Lower Bound | Upper Bound |
| 1 | (Constant) | | 2.147 | .013 |  | 159.672 | .000 | 2.121 | 2.174 |
|  | Problematic Sleep Index (higher=better sleep) | | -.221 | .017 | -.021 | -12.888 | .000 | -.255 | -.188 |
|  | Healthy Diet Score  (higher=better diet) | | -.007 | .002 | -.007 | -4.392 | .000 | -.010 | -.004 |
| 2 | (Constant) | | 1.369 | .025 |  | 54.711 | .000 | 1.320 | 1.418 |
|  | Problematic Sleep Index (higher=better sleep) | | .018 | .019 | .002 | .931 | .352 | -.019 | .054 |
|  | Healthy Diet Score  (higher=better diet) | | -.010 | .002 | -.010 | -5.852 | .000 | -.013 | -.006 |
|  | Age | | .001 | .000 | .010 | 6.368 | .000 | .001 | .002 |
|  | Sex (F=0/M=1) | | -.127 | .004 | -.055 | -33.139 | .000 | -.135 | -.120 |
|  | BMI | | .019 | .000 | .077 | 45.795 | .000 | .018 | .019 |
|  | MH Symp. (higher=worse) | | -.002 | .001 | -.006 | -3.189 | .001 | -.003 | -.001 |
|  | Health Rating (higher=worse) | | .040 | .003 | .025 | 13.896 | .000 | .034 | .045 |
|  | SES  (higher=least affluent) | | .008 | .001 | .020 | 12.490 | .000 | .007 | .009 |

*Model 1: F*(2, 390116)= 93.60, *p≤.*001, *R^2^*(adjusted) = 0.000, *Cohen’s f ^2^*= 0.000

*Model 2: F*(8, 390116)= 513.37, *p≤.*001, *R^2^*(adjusted) = 0.010, *Cohen’s f ^2^*= 0.010

**Table 6**. Regression analysis summary for neutrophil count

| Model | | Unstandardized Coefficients | | Standardized Coefficients | t | Sig. | 95.0% Confidence Interval for B | |
| --- | --- | --- | --- | --- | --- | --- | --- | --- |
|  |  | B | Std. Error | Beta |  |  | Lower Bound | Upper Bound |
| 1 | (Constant) | 4.935 | .016 |  | 301.237 | .000 | 4.903 | 4.967 |
|  | Problematic Sleep Index (higher=better sleep) | -.701 | .021 | -.053 | -33.481 | .000 | -.742 | -.660 |
|  | Healthy Diet Score  (higher=better diet) | -.104 | .002 | -.086 | -53.885 | .000 | -.108 | -.101 |
| 2 | (Constant) | 2.966 | .030 |  | 98.306 | .000 | 2.907 | 3.026 |
|  | Problematic Sleep Index (higher=better sleep) | -.020 | .023 | -.002 | -.870 | .384 | -.064 | .025 |
|  | Healthy Diet Score  (higher=better diet) | -.082 | .002 | -.067 | -41.563 | .000 | -.086 | -.078 |
|  | Age | .003 | .000 | .018 | 11.165 | .000 | .003 | .004 |
|  | Sex (F=0/M=1) | -.011 | .005 | -.004 | -2.423 | .015 | -.020 | -.002 |
|  | BMI | .029 | .000 | .096 | 58.154 | .000 | .028 | .030 |
|  | MH Symp. (higher=worse) | -.002 | .001 | -.004 | -2.062 | .039 | -.003 | .000 |
|  | Health Rating (higher=worse) | .232 | .003 | .118 | 67.460 | .000 | .225 | .238 |
|  | SES  (higher=least affluent) | .018 | .001 | .038 | 24.011 | .000 | .017 | .019 |

*Model 1: F*(2, 390118)= 2041.32, *p≤.*001, *R^2^*(adjusted) = 0.010, *Cohen’s f ^2^*= 0.010

*Model 2: F*(8, 390118)= 2048.08, *p≤.*001, *R^2^*(adjusted) = 0.040, *Cohen’s f ^2^*= 0.041

**Table 7**. Regression analysis summary for basophil count

| Model | | Unstandardized Coefficients | | Standardized Coefficients | t | Sig. | 95.0% Confidence Interval for B | |
| --- | --- | --- | --- | --- | --- | --- | --- | --- |
|  |  | B | Std. Error | Beta |  |  | Lower Bound | Upper Bound |
| 1 | (Constant) | .041 | .001 |  | 68.661 | .000 | .040 | .042 |
|  | Problematic Sleep Index (higher=better sleep) | -.008 | .001 | -.018 | -11.029 | .000 | -.010 | -.007 |
|  | Healthy Diet Score  (higher=better diet) | .000 | .000 | -.011 | -6.582 | .000 | -.001 | .000 |
| 2 | (Constant) | .028 | .001 |  | 25.090 | .000 | .026 | .030 |
|  | Problematic Sleep Index (higher=better sleep) | .001 | .001 | .002 | 1.078 | .281 | -.001 | .003 |
|  | Healthy Diet Score  (higher=better diet) | -.001 | .000 | -.013 | -7.630 | .000 | -.001 | .000 |
|  | Age | -6.018E-5 | .000 | -.009 | -5.786 | .000 | .000 | .000 |
|  | Sex (F=0/M=1) | -.004 | .000 | -.042 | -25.187 | .000 | -.005 | -.004 |
|  | BMI | .000 | .000 | .021 | 12.456 | .000 | .000 | .000 |
|  | MH Symp. (higher=worse) | -2.945E-5 | .000 | -.002 | -1.053 | .292 | .000 | .000 |
|  | Health Rating (higher=worse) | .003 | .000 | .040 | 22.372 | .000 | .003 | .003 |
|  | SES  (higher=least affluent) | .000 | .000 | .025 | 15.589 | .000 | .000 | .000 |

*Model 1: F*(2, 390118)= 83.64, *p≤.*001, *R^2^*(adjusted) = 0.000, *Cohen’s f ^2^*= 0.000

*Model 2: F*(8, 390118)= 245.28, *p≤.*001, *R^2^*(adjusted) = 0.005, *Cohen’s f ^2^*= 0.005

**Table 8**. Regression analysis summary for CRP levels

| Model | | Unstandardized Coefficients | | | Standardized Coefficients | t | Sig. | 95.0% Confidence Interval for B | |
| --- | --- | --- | --- | --- | --- | --- | --- | --- | --- |
|  |  | B | | Std. Error | Beta |  |  | Lower Bound | Upper Bound |
| 1 | (Constant) | | 5.317 | .051 |  | 105.258 | .000 | 5.218 | 5.416 |
|  | Problematic Sleep Index (higher=better sleep) | | -3.192 | .065 | -.080 | -49.440 | .000 | -3.319 | -3.066 |
|  | Healthy Diet Score  (higher=better diet) | | -.182 | .006 | -.049 | -30.542 | .000 | -.194 | -.171 |
| 2 | (Constant) | | -4.354 | .091 |  | -47.779 | .000 | -4.532 | -4.175 |
|  | Problematic Sleep Index (higher=better sleep) | | -.740 | .068 | -.018 | -10.806 | .000 | -.874 | -.606 |
|  | Healthy Diet Score  (higher=better diet) | | -.134 | .006 | -.036 | -22.652 | .000 | -.146 | -.123 |
|  | Age | | .027 | .001 | .051 | 32.000 | .000 | .025 | .029 |
|  | Sex (F=0/M=1) | | -.521 | .014 | -.060 | -37.298 | .000 | -.548 | -.493 |
|  | BMI | | .197 | .001 | .217 | 133.064 | .000 | .194 | .200 |
|  | MH Symp. (higher=worse) | | -.019 | .002 | -.014 | -8.450 | .000 | -.024 | -.015 |
|  | Health Rating (higher=worse) | | .554 | .010 | .093 | 53.426 | .000 | .534 | .574 |
|  | SES  (higher=least affluent) | | .041 | .002 | .029 | 18.317 | .000 | .037 | .046 |

*Model 1: F*(2, 383068)= 1713.638, *p≤.*001, *R^2^*(adjusted) = 0.009, *Cohen’s f ^2^*= 0.009

*Model 2: F*(8, 383068)= 4045.064, *p≤.*001, *R^2^*(adjusted) = 0.078, *Cohen’s f ^2^*= 0.085

**Table 9**. Regression analysis summary for NLR

| Model | | Unstandardized Coefficients | | Standardized Coefficients | t | Sig. | 95.0% Confidence Interval for B | |
| --- | --- | --- | --- | --- | --- | --- | --- | --- |
|  |  | B | Std. Error | Beta |  |  | Lower Bound | Upper Bound |
| 1 | (Constant) | 2.624 | .015 |  | 177.489 | .000 | 2.595 | 2.653 |
|  | Problematic Sleep Index (higher=better sleep) | -.238 | .019 | -.020 | -12.580 | .000 | -.275 | -.201 |
|  | Healthy Diet Score  (higher=better diet) | -.053 | .002 | -.049 | -30.494 | .000 | -.057 | -.050 |
| 2 | (Constant) | 2.143 | .027 |  | 78.016 | .000 | 2.089 | 2.197 |
|  | Problematic Sleep Index (higher=better sleep) | -.064 | .021 | -.005 | -3.116 | .002 | -.105 | -.024 |
|  | Healthy Diet Score  (higher=better diet) | -.033 | .002 | -.031 | -18.697 | .000 | -.037 | -.030 |
|  | Age | .004 | .000 | .028 | 17.292 | .000 | .004 | .005 |
|  | Sex (F=0/M=1) | .191 | .004 | .075 | 45.443 | .000 | .183 | .200 |
|  | BMI | -.012 | .000 | -.044 | -26.319 | .000 | -.013 | -.011 |
|  | MH Symp. (higher=worse) | -.003 | .001 | -.007 | -3.768 | .000 | -.004 | -.001 |
|  | Health Rating (higher=worse) | .149 | .003 | .084 | 47.533 | .000 | .142 | .155 |
|  | SES  (higher=least affluent) | .001 | .001 | .003 | 2.084 | .037 | .000 | .003 |

*Model 1: F*(2, 390113)= 550.280, *p≤.*001, *R^2^*(adjusted) = 0.003, *Cohen’s f ^2^*= 0.003

*Model 2: F*(8, 390113)= 786.135, *p≤.*001, *R^2^*(adjusted) = 0.016, *Cohen’s f ^2^*= 0.016
